# In vivo Wnt pathway inhibition of human squamous cell carcinoma growth and metastasis in the chick chorioallantoic model

**DOI:** 10.1186/s40463-016-0140-8

**Published:** 2016-04-26

**Authors:** Shannon F. Rudy, J. Chad Brenner, Jennifer L. Harris, Jun Liu, Jianwei Che, Megan V. Scott, John Henry Owen, Christine M. Komarck, Martin P. Graham, Emily L. Bellile, Carol R. Bradford, Mark EP Prince, Thomas E. Carey

**Affiliations:** Department of Otolaryngology/Head Neck Surgery, University of Michigan School of Medicine, Ann Arbor, MI 48109-5616 USA; Comprehensive Cancer Center, University of Michigan School of Medicine, Ann Arbor, MI 48109-5616 USA; Cancer Center Biostatistics Core, University of Michigan School of Medicine, Ann Arbor, MI 48109-5616 USA; Genomics Institute of the Novartis Research Foundation, 10675 John Jay Hopkins Drive, San Diego, CA 92121 USA; Department of Otolaryngology/Head Neck Surgery, Stanford University, Stanford, CA 94305 USA; Wayne State University School of Medicine, Detroit, MI 48201 USA

**Keywords:** In vivo cancer model, WNT pathway inhibition, WNT974, Human squamous cell carcinoma, Chorioallantoic membrane, UM-SCC, Cell lines, NOTCH1 mutation

## Abstract

**Background:**

Head and neck squamous cell carcinoma (HNSCC) is an aggressive cancer with poor overall survival. New therapeutic strategies that target specific molecular lesions driving advanced disease are needed. Herein we demonstrate the utility of the chicken chorioallantoic membrane (CAM) assay for in vivo human HNSCC tumor growth and metastasis and the tumor suppressive effects of a new chemotherapeutic agent.

**Methods:**

We tested anti-metastatic effects of a WNT pathway inhibitor, WNT974 (also known as LGK974), which targets porcupine (PORCN) the palmityl-transferase that is essential for secretion of Wnt proteins. CAM assays were performed with 8 HNSCC cell lines: UM-SCC-1, UM-SCC-10A, UM-SCC-10B, UM-SCC-11A, UM-SCC-14A UM-SCC-17A, UM-SCC-17B, UM-SCC-25, and UM-SCC-34.

**Results:**

UM-SCC-1 (University of Michigan Squamous Cell Carcinoma cell line) CAM xenografts contain CD44+ and ALDH+ cancer stem cell (CSC) proportions similar to UM-SCC-1 mouse xenografts supporting the applicability of the CAM assay for study of CSCs. Inhibition of WNT signaling by the *PORCN* inhibitor WNT974 reduced metastatic spread of UM-SCC cells, especially in UM-SCCs with Notch1 deficiency.

**Conclusions:**

Our data demonstrate decreased tumor growth and metastases in tumors from cell lines that showed in vitro responses to WNT974, providing evidence that this agent may have a role in future HNSCC therapy.

## Background

Head and neck squamous cell carcinoma (HNSCC) is the sixth most common form of cancer worldwide [1]. Despite therapeutic strides made in the field of oncology in recent years, the prognosis of HNSCC remains very poor, in large part due to the highly invasive nature of this cancer which often results in extensive local invasion, early dissemination into regional lymph nodes, and metastatic spread of the disease [2]. At the time of diagnosis, two-thirds of patients already have locoregionally advanced disease, defined as Stage 3 or 4. The overall five year overall survival rates for all stages of larynx and oral and pharynx cancer ranges from 63–66 percent respectively [3]. For patients with advanced disease the introduction of adjuvant concurrent chemoradiotherapy has improved survival by roughly 12 %, which corresponds to an improvement in overall 5 year survival from 45 % in 1973 to 53.2 % in 2005 [4]. Given these considerations, there is a clear need for therapeutic advances in this field, and those that will have the most meaningful impact are likely to come in the form of modalities that treat the genetic drivers of advanced-staged disease [5].

The Wnt signaling pathway is an appealing target, as this developmental pathway has been implicated in a large number of human cancers, with recent evidence for an oncogenic role in HNSCC [6]. Deregulation of Wnt signaling from mutation or abnormal expression of pathway components has been implicated to play a role in invasive growth patterns in HNSCC [7]. Normally, the Wnt signal transduction pathway leads to activation of pathways regulating the cytoskeleton, cellular calcium levels and beta-catenin protein expression and transcriptional activation (reviewed in [8–13]). Briefly, the pathway is activated by either autocrine or paracrine signaling through a combination of up to nineteen different Wnt ligands acting on Frizzled receptors. In the absence of Wnt receptor activation, two scaffolding tumor suppressor proteins in the beta-catenin destruction complex called adenomatous polyposis coli (APC) and axin bind to beta-catenin enabling the kinases CK1 and glycogen synthase kinase 3 to sequentially phosphorylate the amino terminus of beta-catenin. The resulting phosphorylated footprint targets beta-catenin for proteasomal degradation. Once activated by Wnt, the Frizzled receptors inhibit the destruction complex through incompletely understood mechanisms. Together, this leads to an accumulation of β-catenin protein, which can then translocate to the nucleus to form a complex with LEF/TCF proteins to regulate transcription of proliferation associated genes.

A small molecule inhibitor of the Wnt-pathway, LGK974, was recently described [14]. The drug name LGK974 has been renamed and is now synonymous with the name WNT974. WNT974 is the name that will be used to reference this compound in this manuscript. WNT974 compound is a potent small molecule inhibitor of Porcupine (PORCN), a membrane-bound O-acyltransferase that adds a palmityol group to Wnt ligands. This modification is essential for Wnt secretion and Frizzled activation. In vitro studies showed a potent inhibitory effect of WNT974 on Wnt signaling, as evidenced by decreased expression of downstream target genes, such as *AXIN2*, as well as reduced Wnt-dependent phosphorylation of LRP6. Of the 96 HNSCC cell lines analyzed, 31 demonstrated a pharmacodymanic (PD) *AXIN2* mRNA reduction response and were thus considered as cell lines that were responsive to treatment with WNT974. Interestingly, there was an enriched rate of response to WNT974 among head and neck cancer cell lines with Notch1 loss-of-function (LOF) mutations [14].

Like Wnt, Notch is also a developmental pathway gene that has recently been implicated in HNSCC tumorigenesis [15–17]. While Notch gain-of-function mutations have been demonstrated in T-cell leukemias and some other forms of cancer, a tumor suppressor role for the Notch pathway has also been suggested in a number of human cancers, including in HNSCC, in which *NOTCH1* LOF mutations were found in 10–15 % of tumors and abnormalities of the Notch pathway in 66 % of patients [18]. The Notch pathway has been proposed to have an inhibitory effect on Wnt signaling in some cell types [19, 20] with evidence suggesting that activated Notch1 signaling suppresses β-catenin signaling in cells that should normally undergo differentiation from the basal layer of the epidermis [17]. With these considerations in mind, we designed an experiment to test the effectiveness of Wnt pathway inhibition with WNT974 on in vivo tumor growth and distant metastasis using the chick chorioallantoic membrane (CAM) assay and human squamous cell carcinoma cell lines. The chick CAM is a multilayered epithelium that consists of ectoderm, mesoderm, and endoderm, as well as extracellular matrix proteins such as laminin and type I collagen, a composition that mimics the tumor environment in humans [21]. Consequently, CAM assays are a well-established in vivo model that has been used to study angiogenesis and tumor invasion in several types of human cancer, including prostate carcinoma, glioma, and bowel cancer [22–24]. Here, we sought to further illustrate the feasibility for study of head and neck cancer cell lines using the CAM assay, determine if HNSCC cancer stem cells (CSCs) can be identified and isolated from primary tumors grown on the CAM, and test the hypothesis that UM-SCC cell line CAM xenograft tumor growth and metastasis can be impaired by WNT974.

## Methods

The aim of this study was to determine the feasibility of the chicken chorioallantoic membrane assay for assessing in vivo tumor response to a novel WNT pathway inhibitor, WNT974, in human head and neck cancer cell lines.

### Ethics consent and permissions

The UM-SCC-1, -10A, -10B, -11A, 14A -17A, -17B, -25, and -34 cell lines were derived in our laboratory from human head and neck tumor explants taken during surgical resection from patients treated at the University of Michigan.The cell line donor-patients gave written informed consent for the use of their tissue to create cell lines in studies reviewed and approved by the University of Michigan Medical School (Ann Arbor) IRBMED institutional review board.

#### Cell lines

The cell lines have been carefully characterized in our laboratory for HNSCC characteristics and each has been genotyped at a minimum of 10 microsatellite markers (Profiler Plus, Invitrogen) to confirm their unique origin [25]. Cell lines with the same number and a letter, i.e. were from the same donor and were derived from primary and recurrent (UM-SCC 10A and UM-SCC-10B) or primary and metastatic (UM-SCC-17A, UM-SCC-17B), lesions respectively. Cells were cultured in Dulbecco modified Eagle medium (Gibco, Life Technologies) containing 2 mM L-glutamine, 1 % nonessential amino acids, 1 % penicillin-streptomycin (Invitrogen), and 10 % fetal bovine serum in a humidified atmosphere of 5 % carbon dioxide at 37 °C. All cell lines were tested for mycoplasma using the MycoAlert Detection Kit (Cambrex) to ensure that they were free from contamination prior to use in these experiments.

The use of fertilized chicken eggs is exempt from vertebrate animal use approval.

#### Chick Chorioallantoic Membrane (CAM) Assay

Fertilized white leghorn chicken eggs were obtained from Charles River Labs (Norwich, CT). The use of fertilized chicken eggs is exempt from vertebrate animal use approval. The eggs were kept in an incubator at 99.5 degrees Fahrenheit at a humidity range of 45–55 %. Eight days following arrival, the embryos were assessed for viability, performed by trans-illumination of the egg in a dark room to identify the presence of an embryo and surrounding blood vessels. A 1 cm^2^ window was drawn on the egg shell overlying the most vascularized area of each viable embryo. Two small holes were then bored into the egg shell, one in the center of the window and the other at the apex of the egg, overlying a naturally occurring air pocket. A rubber pipette bulb was used to suction a small amount of air out of this apical pocket, causing the chorioallantoic membrane to drop downward, away from the vent hole in the drawn window. The window was then opened using a Dremel 1100-N/25 7.2-Volt Stylus Lithium-Ion Cordless Rota Drill (Robert Bosch Tool Company) and was covered with a piece of clear adhesive tape to protect the embryo and prevent dehydration. This window served as the site for subsequent cancer cell inoculation in our preliminary experiment, and cancer cell and drug inoculation in our WNT974 treatment experiment (See Liu, Min et al Translational Oncology 6:273-281, 2013 for illustrations of the model).

Seven days after the administration of cancer cells, the chick eggs were opened with sterile scissors, and primary tumors were dissected out and weighed. The chick embryos were then dissected, removing the lungs and livers which were immediately placed on ice in labeled 10 mL conical containers. The samples were stored in the -80 degree C freezer until DNA extraction.

#### DNA extraction and analysis

Chick embryo livers and lungs were thawed, rinsed with 5 mL PBS and homogenized with a handheld homogenizer (Omni International) using a sterile tip for each sample. DNA extraction was performed using the QIAgen DNeasy Blood & Tissue Kit (QIAGEN Group), following the manufacturer’s specifications. Purified DNA was quantified using a spectrophotometer, adjusted to a concentration of 0.2ug/uL, and stored at -20 degrees C. Quantitative PCR (qPCR) was performed on the DNA samples using primers specific for a human Alu sequence. Alu sequences are primate-specific, and thus their detection in chick organs represents disseminated human cells, or cancer metastases. The copy threshold, or CT, values of the liver and lung specimens from the eight HNSCC cell lines were compared to the CT values of the negative controls of the respective organ. Student T-tests were used to compare the CT values of both organs of each cell line to the respective negative control.

#### Applicability assay

The initial experiments were designed to demonstrate feasibility of the CAM assay for use with HNSCC cancer cells using eight cell lines (UM-SCC-1, -10A, -10B, -11A, -17A, -17B, -25, and -34)..The cells were grown in 150 cm^2^ plastic flasks, trypsinized, counted, and resuspended in a mixture of 10 % matrigel (BD Biosciences) and 90 % DMEM for a total volume of 30uL per eggs such that each egg received 2.5 million cancer cells. The prepared chick eggs were removed from the incubator, and the cancer cell suspension was laid on top of the chorioallantoic membrane using a 100uL pipette tips. For each cell line, five eggs were each inoculated. An additional ten eggs received a 30uL suspension of 10 % matrigel and 90 % DMEM with no cancer cells to serve as treatment, embryo viability, and specificity controls.

#### Flow cytometry

UM-SCC-1 cancer cells were administered to an additional ten eggs for cancer stem cell analysis. Seven days following cancer cell administration, primary tumors were dissected from eight viable embryos and placed on ice in 15 mL tubes filled with DMEM media. Tumor tissue was minced and digested in DMEM/F12 (Gibco) with 1x collagenase/hyaluronidase (Stem Cell Technologies). After two hours of digestion, the mixture was strained through a 40 μm sieve and the cells were counted before being prepared for flow cytometry. CD44 expression was detected using an APC-conjugated antibody (BD Pharmingen). Aldehyde dehydrogenase (ALDH) expression was detected using the ALDEFLUOR kit (StemCell Technologies). The aldefluor substrate freely diffuses into cells and reacts with human aldehyde dehydrogenase enzyme, producing a fluorescent reaction product that is proportional to the enzymatic activity. This reaction does not occur in the chicken host cells.

Tumor cells from the primary tumors of the eight viable chick embryos were pooled together to gather sufficient cells for an adequate assessment of CSCs in the tumors grown on the CAM. Fluorescence-activated cell sorting gates were established for ALDH expression using the inhibited control (DEAB) along the fluorescein isothiocyanate (FITC) channel with excitation and emission wavelengths of approximately 495 nm/521 nm. For CD44 expression, cell sorting gates were established using the APC-conjugated isotype control along the allophycocyanin (APC) channel with excitation and emission wavelengths of approximately 650 nm/660 nm.

### WNT974 treatment assay

To test the effect of Wnt pathway inhibition on tumor growth and distant metastasis we first performed a dose-finding assay with WNT974 to assess for potential toxicity of this pathway inhibitor on chick embryos. Three drug concentrations were tested, 0.1 μM, 0.31 μM, and 1 μM. μM. For each drug concentration, five chick eggs were dosed every other day for a total of four doses. An additional five eggs were treated with 5uL DMSO (the WNT974 vehicle) to control for vehicle toxicity to the embryos. This assay showed minimal toxicity of these drug concentrations on the chick embryos, with all five embryos given 1 μM WNT974 remaining viable and phenotypically normal through harvesting. Thus 1 μM was selected as the treatment dose. Four UM-SCC cell lines were selected to test the effect of WNT974 on tumor growth on the CAM and on distant metastases to liver and lung. Two cell lines, UM-SCC-11A and -25, were shown via deep exome sequencing to have *Notch1* mutations (UM-SCC-25 contains a nonsense LOF mutation, and UM-SCC-11A contains a missense mutation), and both exhibited a pharmacodynamic (PD) change in *AXIN2* mRNA expression in response to WNT974 during the in vitro analysis performed by GNF. A third cell line, UM-SCC-1, containing wildtype *NOTCH1*, also showed a PD response to WNT974. The fourth line, UM-SCC-14A, also wildtype for *NOTCH1,* was a PD non-responder in vitro. The CAM assay was prepared as described above. For each cell line, 2.5 million cells were administered to each of 32 eggs (performed in separate assays per cell line due to limited incubator space). Of these, half served as treatment eggs and the other half were vehicle control eggs. The treatment arm received 5 μL of 1 μM WNT974 once daily, every other day starting the day of cancer cell inoculation for a total of four doses. The control arm received 5uL DMSO every other day for a total of four doses. There were also 5 manipulation-only control eggs per cell line, which were injected with 30 μL of 10 % matrigel and 90 % DMEM but no cancer cells. Eggs were opened seven days following cancer cell administration, all identifiable primary tumors dissected and weighed, the lung and liver tissues harvested and DNA was purified as described above.

#### Statistical analysis

An *a priori* power calculation was performed. Given a Type I error rate of 0.05 and power of approximately 85 %, it was determined that a sample size of at least 9 tumors in each treatment group was needed to detect a treatment effect size of 1.5 standard deviations in magnitude. For each of the four cell lines, tumor weights for the treatment and non-treatment groups were recorded and compared using two-tailed T-tests. Given the need to run the assay twice for each cell line due to space limitations, an ANOVA model for each cell line was used to evaluate treatment effect controlling for assay batch as a main effect and as potential effect modifier in the model parameterization. These analyses revealed no significant batch effect in any of the four cell lines, thus allowing pooling of the data from both batches. The qPCR cycle threshold (CT) values for both the liver and lung specimens in the treatment group for each cell line were compared to the CT values of the corresponding organ and cell line using a two-tailed T-test, generating a P-value for both the liver and lungs in each cell line. P-values <0.05 were considered statistically significant.

## Results

### UM-SCC cell lines grow and metastasize in CAM assays

**Fig. 1:**
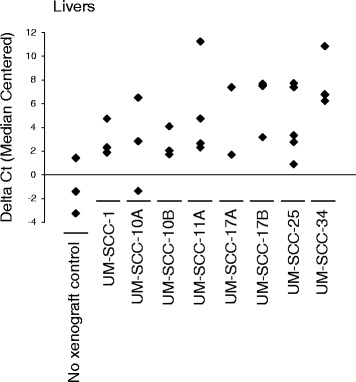
CAM xenografted UM-SCC cell lines metastasize to the liver. This figure compares the relative content human DNA in the tissue as determined by amplified human Alu DNA sequences. The values were calculated by comparing the Alu qPCR cycle threshold (Ct) of a given liver specimen to the organs of non-xenografted control animals. Each qPCR reaction was completed in quadruplicate. Error bars are standard error of the mean

Five chick embryos were implanted with cancer cells for each of the eight UMSCC cell lines tested in this study. For each cell line, the number of viable embryos examined for tumor growth is given following the cell line: UM-SCC-1: 5, UM-SCC-10A: 3, UM-SCC-10B: 4, UM-SCC-11A: 5, UM-SCC-17A: 3, UM-SCC-17B: 5, UM-SCC-25: 5, and UM-SCC-34: 4 embryos. Of the viable embryos, a subset was randomly chosen for primary tumor dissection and weighing. Tumor weights ranged from 27.4 to 82.4 mg with consistency within cell lines (all weights within two fold of one another) and less consistency between cell lines (up to a fourfold difference in tumor weights). To assess the metastatic ability of these cell lines in this assay, quantitative PCR performed on DNA isolated from the livers of xenograft bearing animals and negative control specimens are shown in Fig. 1*.* This demonstrated that all eight of the cell lines metastasized to the livers of the developing chicks. qPCR was also performed on DNA isolated from the chick lungs. However, only one negative control was included in the qPRC analysis due to the initial poor egg viability, and therefore these data have not been included in Fig. 1.

#### CAM assays as a cancer stem cell model

**Fig. 2:**
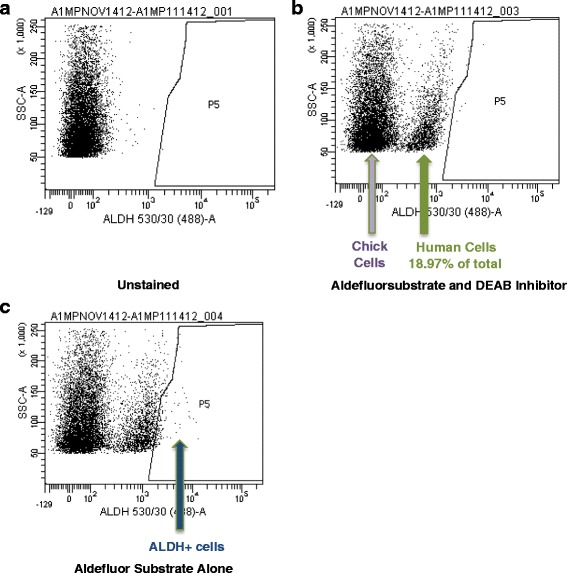
Analysis of ALDH positive cells in UM-SCC-1 CAM xenografts. **a** Flow cytometry of unstained sample of UM-SCC-1 primary tumor cells grown in the CAM assay. **b** Flow cytometry of UM-SCC-1 CAM xenograft tumor cells stained with Aldefluor substrate and DEAB inhibitor. The Aldefluor substrate only reacts with the mammalian ALDH enzyme, so inclusion of the substrate will allow the human cells to shift forward while the chick cells will show no shift from the unstained sample. **c** Removal of the DEAB inhibitor results in a right-shift of the ALDH+ population. 1.55 % of the total cell population is ALDH+, but only 18.97 % of the total cell population is human cells. Therefore, 8.17 % of the UM-SCC-1 cells in the CAM xenograft were analyzed to be ALDH+

**Fig. 3:**
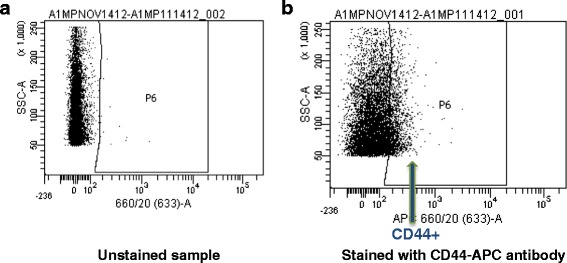
Analysis of CD44 positive cells in UM-SCC-1 CAM xenografts. **a** Flow cytometry results of unstained sample of UM-SCC-1 primary tumor cells grown in the CAM assay. **b** Flow cytometry results of UM-SCC-1 primary tumor cells stained with CD44-APC antibody. 15.44 % of the total cell population is CD44+, but only 18.97 % of the total cell population is human cells. Therefore 81.39 % of the UM-SCC-1 cells in the CAM xenograft are CD44+

Next, we assessed the relative percentage of stem cell markers in tumors excised from the CAM xenografts. Eight independent UM-SCC-1 primary tumors were pooled to insure sufficient cells for stem cell analysis. Flow analysis with the aldefluor substrate and the DEAB inhibitor showed that 18.97 % of the total cell population reacted with the aldefluor substrate and were human cells and 81.03 % consisted of chick (aldefluor fluorescence negative) cells (Fig. 2). Of the human cell population when the ALDH inhibitor DEAB is removed, 8.17 % were ALDH^high^ (also known as the stem cell or side population [26]) and 81.29 % were CD44^high^ (Fig. 3). These results are consistent with what we have previously observed with CSC populations in UM-SCC-1 cells excised from a mouse model [26, 27]. Together, these results indicate that tumors grown in the CAM assay continue to generate HNSCC CSCs at rates similar to those observed in the murine model, thus offering an alternative to murine xenografts as an in vivo approach to study CSCs in HNSCC.

### WNT974 disrupts UM-SCC cell line xenograft growth and metastasis

**Fig. 4:**
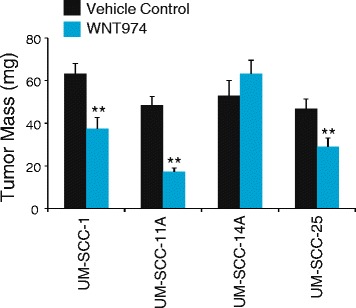
WNT974 blocks UM-SCC-1, -11A and -25, but not -14A, CAM xenograft growth. UM-SCC cell lines were implanted on CAM models and treated with 1 μM WNT974 or vehicle control (DMSO) every other day for eight days. At the conclusion of the experiment, tumors were harvested from the CAM and weighed. We assessed 22, 33, 24 and 27 viable animals UM-SCC-1, -11A, -25 and -14A, respectively, with half receiving vehicle control and the rest WNT974. Averages of all groups are shown along with standard error. ** *P* < 0.05

**Fig. 5:**
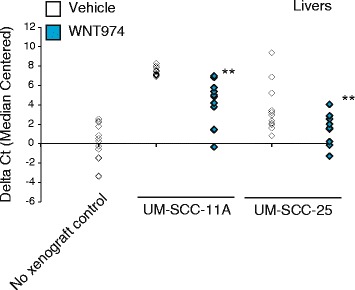
WNT974 blocks liver metastasis of UM-SCC-11A and UM-SCC-25 CAM xenografts. Livers from UM-SCC-11A and -25 CAM xenografts (from Fig. 4) were harvested at the conclusion of the experiment and assessed for human ALU DNA sequences by qPCR. WNT974 was administered every other day to a final concentration of 1 μM. Livers from non-xenografted animals were used as a negative control and Ct difference from the median centered average of normal livers is shown. All qPCR assays were run in quadruplicate. ** *P* < 0.05

After establishing the CAM model using several of the genetically characterized UM-SCC cell lines [14], we sought to test the effect of WNT974 on the growth and metastatic ability of cell lines of varying *NOTCH1* mutation status and in vitro pharmacodynamic response to WNT974: UM-SCC-11A (*NOTCH1* mutant, PD responder), UM-SCC-25 (*NOTCH1* frameshift mutant, PD responder), UM-SCC-1 (*NOTCH1* wildtype, PD responder), and UM-SCC-14A (*NOTCH1* wildtype, PD non-responder). Tumor weight analysis shown in Fig. 4 revealed a statistically significant decrease in primary tumor weight between treated and untreated embryos in UM-SCC-11A (*p* = 1.0 × 10^-7^), UM-SCC-1 (*p* = 0.002), and UM-SCC-25 (*p* = 0.0076), the three cell lines that previously showed in vitro pharmacodynamic responses to WNT974 [14]. There was no difference in tumor weights between treated and untreated embryos in UM-SCC-14A, which correspondingly was a non-PD responder. Furthermore, qPCR analysis of organs harvested from developing chicks for human ALU DNA revealed a statistically significant reduction in liver metastases in UM-SCC-11A (*p* = 2.5 × 10^-5^) and UM-SCC-25 (*p* = 0.02) (Fig. 5). Together the data demonstrates that WNT974 can effectively disrupt xenograft tumor growth and liver metastasis of several UM-SCC cell lines.

## Discussion

We and others have used the CAM assay for a variety of studies [24, 26, 27]. Here we confirmed the value of CAM assays as short term xenograft models of HNSCC proliferation and metastatic behavior in vivo with a variety of UM-SCC cell lines. We show that nine different UM-SCC cell lines form tumors and distant metastasis in this system. In fact, the high rates of observed tumors and metastases demonstrate the utility of the chick CAM assay for in vivo study of HNSCC tumor behavior as well as the potential for an expanded use of this preclinical model for studying response to drug therapy in head and neck cancer. Our results support the value of this model in screening tumors with different genomic compositions for response to new drug therapies, which could help expedite the preclinical phase of drug development, as well as to help identify patients who are most likely to respond to new therapies. This study demonstrates the generation of cancer stem cells within primary tumors grown in the CAM assay. Flow cytometric analysis of tumor cells detected CD44^high^ and ALDH^high^ cells at similar rates to those observed in murine model HNSCC tumors. Our ability to detect CSCs on tumor cells grown in the CAM assay provides opportunities for use of this model in the study of HNSCC CSC populations, behavior, and response to drug therapies. The CAM model has several advantages as an adjunct to murine xenografts [24]. The CAM model is relatively inexpensive, can be completed in approximately a 16 day window from egg delivery until harvest and is sensitive to metastatic spread.

In addition to evaluating CAM assays as a model of therapeutic response, we explored the interaction between the Wnt and Notch signaling pathways in HNSCC, specifically in the context of the anti-cancer effects of Wnt pathway inhibition in tumors with different types of Notch pathway mutation. Given the previously proposed inhibitory effect of the Notch pathway on Wnt signaling [19, 20], it is possible that tumors that harbor mutations in Notch1 have increased Wnt signaling and are therefore more susceptible to Wnt pathway inhibition. This is also supported by the early in vitro study of WNT974, which revealed an enriched rate of response to WNT974 among head and neck cancer cell lines with Notch1 loss-of-function (LOF) mutations, suggesting that Notch1 mutation status may play a role in responsiveness to Wnt pathway inhibition. However, it is also possible that Wnt gain of function mutations, which were not tested for in the cell lines used in this study, could be the independent driving force in tumorigenesis in the responder cell lines, resulting in the observed decreased tumor growth and metastasis following Wnt pathway inhibition in three of the four cell lines tested. Lack of sufficient statistical power due to the limited number of HNSCC cell lines tested did not allow for correlation of sensitivity of WNT974 in the CAM model to Notch 1 mutations status. However, the study provides strong, early evidence for a potential role of WNT974 in the treatment of patients with HNSCC as a significant therapeutic response in the CAM assay was seen with three of the four tumor cell lines tested. Future experiments will analyze the effects of WNT974 in the context of additional biomarkers as well as in combination with other agents in order to predict and improve clinical response.

## Conclusion

HNSCC cell line xenograft growth, cancer stem cell distribution, metastasis and therapeutic response can be effectively assessed in CAM assays in a manner consistent with mouse xenograft assays. This allows for an expeditious means to screen the efficacy of new therapeutic agents across a large number of head and neck tumors such that more time consuming mouse xenograft models can be focused on the precision medicine protocols most likely to advance to clinical trials. Therefore, we expect that implementation of this model will significantly reduce the pre-clinical phase of drug development timelines.
